# HELLP综合征1例并文献复习

**DOI:** 10.3760/cma.j.cn121090-20241130-00507

**Published:** 2024-12

**Authors:** 新 张, 红彬 孟, 荣祎 曹, 丽瑛 蔡

**Affiliations:** 哈尔滨医科大学附属第一医院产科，哈尔滨 150001 Obstetrics Department of the First Affiliated Hospital of Harbin Medical University, Harbin 150001, China

## Abstract

HELLP综合征，也称为溶血、肝酶升高和血小板减少综合征，是与高血压相关的严重妊娠期并发症，与母儿不良预后有关。尽管在临床工作中产科医师对该病的认识逐渐完善，但部分HELLP综合征患者因其发病急、进展迅速，在临床诊治过程中仍然存在困难与挑战。本文报道1例哈尔滨医科大学附属第一医院救治的HELLP综合征患者，结合文献复习为临床诊治提供思路。

## Abstract (en)

HELLP syndrome, also known as hemolytic syndrome, elevated liver enzymes, and thrombocytopenia syndrome, is a severe pregnancy complication associated with hypertension and is associated with poor maternal and child outcomes. Although the understanding of the disease by the obstetrician has been gradually improved in the clinical work, some patients with HELLP syndrome still have difficulties and challenges in the process of clinical diagnosis and treatment due to their rapid onset and rapid progress. This paper reports a case of HELLP syndrome treated in the First Affiliated Hospital of Harbin Medical University, hoping to provide ideas for the clinical diagnosis and treatment of the literature review.

HELLP综合征即微血管内溶血、肝酶升高和血小板减少，是妊娠期严重的并发症，首发症状可能出现右上腹疼痛、恶心或呕吐等非特异症状，需与妊娠期急性脂肪肝、血栓性血小板减少性紫癜、系统性红斑狼疮和抗磷脂综合征相鉴别。HELLP综合征往往发生于妊娠晚期，本例发生于妊娠36周+2，起病急骤，进展迅速，急诊终止妊娠后转入重症监护病房（ICU）治疗。本文将对其救治过程进行描述并结合相关文献展开讨论。

## 病例资料

患者，女，40岁，因“停经36周+2，恶心呕吐伴上腹痛8 h”于2021年6月7日6时19分收入产科。患者于停经32周出现血压升高伴双下肢水肿，血压最高达158/92 mmHg（1mmHg = 0.133kPa），口服“拉贝洛尔”（具体用药剂量不详）后未监测血压。8 h前出现恶心、呕吐，伴上腹痛，2 h前出现不规律下腹痛，遂就诊我院，给予硫酸镁10 g解痉（1～2 g/h）、盐酸尼卡地平降压、地塞米松6 mg肌肉注射。既往史：左髌骨骨折手术史、剖宫产手术史。入院查体：体温36.6 °C、脉搏74次/min、呼吸18次/min、血压210/112 mmHg、体重80 kg，身高160 cm、体质量指数（BMI）31.25 kg/m²。一般状态欠佳，皮肤、巩膜黄染、肉眼血尿；规律宫缩，少量阴道流血，无阴道流液，双下肢水肿（++）。入院当日辅助检查：血常规：WBC 17.73×10^9^/L、HGB 139 g/L、PLT 82×10^9^/L；凝血项标本溶血，复查后再次溶血；其他检验结果均因标本溶血未回报。产科超声：妊娠34～35周，子宫前壁下段肌壁菲薄，盆腔积液；胎心监测：130～140次/min，可见频繁减速，最低达60 次/min；心脏彩超：二尖瓣少量反流、左室舒张功能减低；肝胆胰脾超声：脂肪肝、餐后胆囊、腹腔肠管胀气；初步诊断：重度子痫前期、妊娠合并血小板减少、妊娠合并子宫瘢痕、胎儿宫内窘迫、妊娠合并肥胖、高龄经产、妊娠36周+2，孕2产1，头位，单胎妊娠。入院后解痉降压治疗的同时，积极进行术前准备。

6月7日7时40分腰麻联合硬膜外麻醉满意后入腹，腹腔内可见淡黄色积液约1 500 ml，膀胱与子宫前壁大面积致密粘连，瘢痕菲薄，组织糟脆，可见浆膜层下的羊水及胎儿毛发。破膜后见羊水量少、墨绿色，以枕左前位娩出胎儿。胎盘母体面可见陈旧性凝血块（面积约2 cm×2 cm）。术中子宫收缩不良，予卡前列素氨丁三醇注射液250 µg肌注+子宫加压缝合术止血，见宫腔内持续性渗血（深褐色稀薄不凝），考虑凝血功能障碍，给予宫腔填塞纱布压迫止血，放置腹腔引流管1枚、腹直肌表面负压引流管1枚。逐层缝合后，再次肌注卡前列素氨丁三醇注射液250 µg促宫缩，复查血常规、凝血项。术中出血400 ml，血性尿液200 ml，输液1 750 ml，输新鲜冰冻血浆380 ml、冷沉淀20 U、血小板1治疗量，输人纤维蛋白原4 g。10时15分手术结束，术后转入ICU治疗。患者肝功回报：丙氨酸氨基转移酶（ALT）705 U/L、天门冬氨酸氨基转移酶（AST）1 897 U/L、总胆红素（TBIL）57.18 µmol/L；LDH 1 295.7 U/L，空腹血糖7.09 mmol/L。入院时凝血项回报：凝血酶原时间（PT）19.1 s、活化部分凝血活酶时间（APTT）31.5 s、纤维蛋白原定量（FIB）1.13 g/L、D-二聚体定量（D-D）29.52 mg/L。术中血常规：HGB 107 g/L、PLT 39×10^9^/L；术中凝血项：PT 18.5 s、APTT 28.6 s、FIB 1.40 g/L、D-D 34.45 mg/L。根据中国弥散性血管内凝血诊断积分系统[1]，患者存在导致弥散性血管内凝血（DIC）的原发病（2分）、PLT 39×10^9^/L（2分）、D-D 34.45 mg/L（3分）、PT 18.5 s（2分），DIC积分为9分，可诊断DIC。术后诊断：HELLP综合征、DIC、重度子痫前期、胎盘早剥、胎儿宫内窘迫、妊娠期糖尿病、妊娠合并肥胖、妊娠合并子宫瘢痕。

予硝普钠50 mg与尼卡地平20 mg持续静点降压、比阿培南（0.3 g，每日2次）抗感染、硫酸镁（10 g，1次/日）预防产后子痫、异甘草酸镁（200 mg，每日1次）+多烯磷脂酰胆碱（931 mg，每日1次）保肝治疗，并继续监测血常规、凝血项。术后10 h患者出现切口渗血，床旁超声检查排除腹腔内出血及肝破裂，继续纠正凝血障碍，术后当天予冷沉淀40 U+血浆730 ml+血小板0.5治疗量+红细胞4 U；术后第1天予冷沉淀20 U+血浆390 ml+血小板1治疗量+红细胞2 U。患者在院期间血常规、凝血项变化如表1。

**表1:** HELLP综合征患者血常规和凝血项检测结果

检测时间	WBC（×10^9^/L）	HGB（g/L）	PLT（×10^9^/L）	PT（s）	APTT（s）	TT（s）	FIB（g/L）	D-D（mg/L）
术中	13.40	107	39	18.5	28.6	25.5	1.40	34.45
术后90 min	15.44	89	58	15.2	25.8	19.8	2.69	22.48
术后10 h	20.79	68	39	15.0	28.1	21.0	2.69	18.07
术后第1天	20.20	74	56	13.0	27.0	18.6	4.10	20.10
术后第2天	27.09	80	80	12.0	23.9	15.5	6.18	7.59
术后第3天	23.83	92	109	11.0	25.9	16.3	4.93	45.21
术后第4天	19.15	107	129	11.0	25.4	16.7	4.79	53.05
术后第5天	14.98	121	108	–	–	–	–	–
术后第6天	9.79	103	132	–	–	–	–	–
参考范围	3.69～9.16	113～151	98～300	9.8～12.1	25～31.3	14～21	1.8～3.5	0～0.55

**注** PT：凝血酶原时间；APTT：活化部分凝血活酶时间；TT：凝血酶时间；FIB：纤维蛋白原定量；D-D：D-二聚体定量；“–”未检测

患者术后第2天肉眼血尿消失，取出宫腔纱布无明显出血；术后第3天拔除腹壁引流管；术后第4天由ICU转入普通病房；术后第8天拔除腹腔引流管。出院时一般状态良好，血压127/87 mmHg，心率72次/min，皮肤巩膜无黄染，子宫收缩良好，切口周围皮肤瘀斑、无异常渗出，阴道少许血性恶露、无异味。ALT 37 U/L、AST 22 U/L、总胆红素10.30 µmol/L，肌酐147.3 µmol/L。

## 讨论并文献复习

HELLP综合征是一种危及生命的妊娠并发症，其发病率为0.5％～0.9％，孕产妇死亡率为0～24％，胎儿死亡率约37％[2]，早期识别、积极治疗对改善母婴结局至关重要。HELLP综合征的病因和机制尚未完全阐明，目前认为是多因素、多系统、多机制共同作用的结果，包括血管内皮损伤、氧化应激异常、免疫炎症过度激活、脂代谢异常、遗传因素等。既往研究[3]认为，HELLP综合征是子痫前期的一种严重表型，因此HELLP综合征可能与胎盘发育和功能障碍有关。

HELLP综合征的诊断主要依据实验室检查，目前存在2种诊断标准：Tennessee分类和Mississippi三阶段分类。Tennessee分类：①微血管病性溶血性贫血伴血涂片异常、血清结合珠蛋白降低和LDH水平升高；②AST或ALT升高至70 IU/L以上，LDH高于600 IU/L或胆红素超过1.2 mg/dl；③PLT低于100×10^9^/L。若只符合其中2条，称为不完全性HELLP综合征（ELLP）[4]。Mississippi分类根据血小板的最低值进行三阶段分类：Ⅰ级（重度）：PLT≤50 000/µl、AST或ALT≥70 IU/L、LDH≥600 IU/L；Ⅱ级（中度）：50 000/µl<PLT≤100 000/µl、AST或ALT≥70 IU/L、LDH≥600 IU/L；Ⅲ级（轻度）：100 000/µl<PLT≤150 000/µl、AST或ALT≥40 IU/L、LDH≥600 IU/L。

HELLP综合征最常见的症状包括蛋白尿、高血压、右上腹痛、恶心呕吐、头痛、视力改变和黄疸，但部分患者无高血压和尿蛋白表现。临床工作中，若患者LDH和转氨酶水平升高、有出血倾向、重度子痫前期，即使PLT和凝血项正常，也应考虑HELLP综合征[5]。HELLP需与其他疾病相鉴别，如抗磷脂抗体综合征、系统性红斑狼疮，可由狼疮抗凝物、抗心磷脂抗体和抗β_2_糖蛋白-Ⅰ抗体区分；妊娠期急性脂肪肝，常发生在妊娠晚期，通常无高血压和蛋白尿，特异性表现为凝血障碍和低血糖；溶血性尿毒症综合征（hemolytic uremic syndrome，HUS）以急性肾功能衰竭为特点，HUS血红蛋白水平较HELLP综合征低，终止妊娠后若实验室指标持续异常需警惕HUS；血栓性血小板减少性紫癜通常表现为重度血小板减少，发病机制与特异性血管性血友病因子裂解蛋白酶ADAMTS13的缺乏相关，检测ADAMTS13活性明显降低、Coombs试验通常阴性、凝血功能一般无紊乱。

本例患者出现明显的恶心、呕吐、上腹痛的表现，皮肤黄染的体征以及采血化验反复溶血，肝功回报后可明确诊断HELLP综合征，而凝血项提示发生DIC，可能与以下原因有关：HELLP综合征广泛的内皮损伤和促凝血因子的释放导致DIC的发生[6]；本例术中发现胎盘早剥，虽然胎盘早剥总失血量较低，但胎盘滋养层细胞完整性的破坏导致组织因子释放到母体循环，激活大量异常凝血级联反应，生成大量凝血酶，促进发展为DIC。相比于产后出血，胎盘早剥导致的DIC发展更迅速、病情更严重[7]。有文献[8]表明，胎盘早剥是HELLP综合征患者发生DIC的独立危险因素，而非与肝功能障碍直接相关。

HELLP综合征的治疗包括终止妊娠及支持治疗。联合产科、麻醉科、ICU、输血科等多学科协作为患者终止妊娠。HELLP并非绝对的剖宫产指征，但剖宫产是终止妊娠最快、缓解HELLP综合征最有效的方法，患者既往存在剖宫产手术史且出现急性胎儿宫内窘迫，需立即剖宫产。若术前PLT<50×10^9^/L，建议输注血小板。术中充分止血，尽量缩短手术时间，根据情况监测血常规、凝血项，输注血制品、纤维蛋白原、氨甲环酸等物质改善凝血紊乱。正常妊娠期间为减少产后出血，纤维蛋白原水平会增加至非孕期的2倍左右，当纤维蛋白原低于3 g/L，需警惕是否存在凝血异常，以便及早补充凝血因子。

终止妊娠后大多数患者的实验室指标可在72 h内有所改善，但仍有个例在终止妊娠后出现转氨酶持续升高、血小板持续下降，因此产后也需持续监测实验室数据，间隔12 h复查包括血常规、肝肾功能等数据，其中血清结合珠蛋白是反映溶血的敏感指标[9]。根据Mississippi分类，该患者为Ⅱ类HELLP综合征，Mississippi Ⅰ类患者中有44％的患者可能出现孕产妇死亡、急性肝衰竭、肺出血、DIC等严重并发症，而Ⅱ类和Ⅲ类患者发生率为13％、23％[10]，因此术后转入ICU十分必要。一般治疗包括解痉、降压、输血液制品和免疫球蛋白、抗凝以及血浆置换[11]，产后应用地塞米松72 h（8 mg，间隔8 h/次）可能增加血小板[12]。当HELLP综合征在产后72 h持续存在时，血浆置换治疗是有益的[13]，接受血浆置换治疗的患者死亡率显著降低，ICU住院时间显著缩短。既往存在HELLP综合征病史的孕产妇，在将来的妊娠中有5％～52％风险再次出现妊娠期高血压疾病[14]。因此，在将来的妊娠过程中，应在孕早期给予干预措施，让患者保持健康的生活方式，尽量降低复发风险。

综上所述，HELLP综合征为产科的危急重症，对母婴产生极大威胁，多学科协作是拥有母婴良好结局的前提，诊疗过程中应关注症状及实验室数据的变化趋势，拥有职业敏感性和对疾病的深刻认知，从而尽早识别、及时处理。
